# Where is the greatest risk of COVID-19 infection? Findings from Germany’s largest public health department, Cologne

**DOI:** 10.1371/journal.pone.0273496

**Published:** 2022-08-24

**Authors:** Lukas Broichhaus, Julian Book, Sven Feddern, Barbara Grüne, Florian Neuhann, Johannes Nießen, Gerhard A. Wiesmüller, Annelene Kossow, Christine Joisten

**Affiliations:** 1 Cologne Health Department, Infektions- und Umwelthygiene, Neumarkt, Köln, Germany; 2 Institute for Occupational Medicine and Social Medicine, University Hospital, Medical Faculty, RWTH Aachen University, Aachen, Germany; 3 Heidelberg Institute of Global Health, Heidelberg University Hospital, Heidelberg, Germany; 4 School of Medicine and Clinical Sciences, Levy Mwanawasa Medical University, Lusaka, Zambia; 5 Institute of Hygiene, University Hospital Muenster, Münster, Germany; 6 Department for Physical Activity in Public Health, Institute of Movement and Neurosciences, German Sport University Cologne, Cologne, Germany; Nanyang Technological University, SINGAPORE

## Abstract

**Background:**

SARS-CoV-2 has been spreading worldwide since late 2019. Before vaccines became available, exclusively non-pharmaceutical measures were used to prevent transmission of infection. Despite the fact that vaccinations are now available, it is still important to identify relevant transmission routes in order to contain the COVID-19- or further pandemics. Therefore, this study aims to systematically analyse data from the largest public health department in Germany to determine the significance of the various known and unknown transmission situations in terms of the proportion of infections.

**Methods:**

All infections in Cologne were systematically recorded by the local health department. In addition to clinical data, the transmission situations were recorded and categorised as pertaining to social contact, work contact, travellers, health care workers, users of educational institutions, visitors of community institutions, infection in the context of medical treatment, and unknown infection.

**Findings:**

The analysis included 25,966 persons. A transmission situation could be identified in 82.7% of the cases (n = 21,477). Most persons (42.1%) were infected due to social contact, primarily within their own household. Another 22.3% were infected at their place of work; this was particularly common among staff members of medical facilities, nursing homes and educational institutions. In 17.3% of the cases, the transmission situation remained unknown; the cases with unknown transmission situation were slightly more often symptomatic (75.2%) than the cases with known transmission situation (69.4%).

**Interpretation:**

Considering that during the study period the leading strains were the wild-type and alpha-variant transmission rather occurred during scenarios involving close contacts than in anonymous situations. Presumably, however, the findings can be transferred to the new variants. Therefore, in order to prevent transmission, besides vaccination regular antigen tests and/or appropriate protective measures remain relevant until this pandemic has subsided.

## Introduction

**Fig 1:**
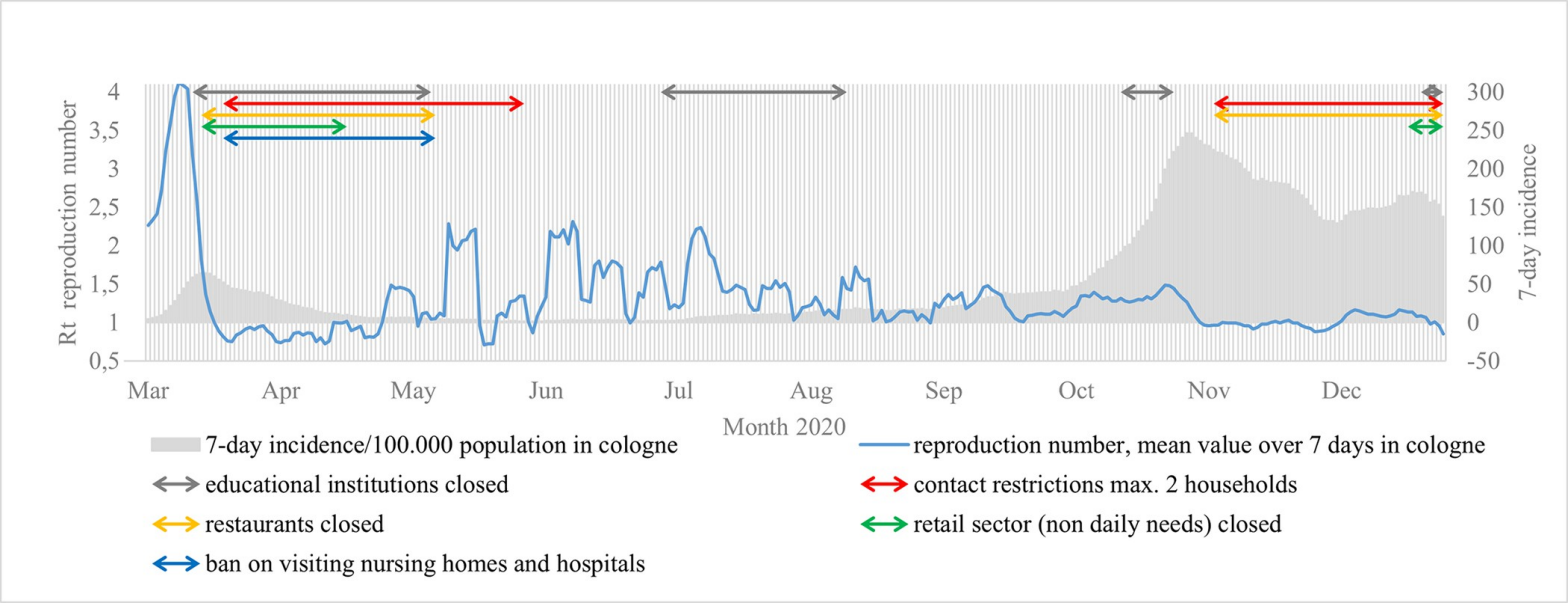
Progression of the pandemic in Cologne with different non-pharmaceutical interventions.

The first cases of SARS-CoV-2 (COVID-19) in Germany occurred in January 2020; by the following month, cases began to be reported in Cologne [1]. Before December 2020, the total number of reported cases was 26,535 (see Fig 1). Since the vaccination drive was not yet underway in Germany at that stage, various non-pharmaceutical, general measures were administratively imposed to contain the pandemic. Such measures included social distancing regulations, wearing protective medical masks and restrictions on private social contact, schools and gastronomy [2] (see Fig 1). In addition, the local health authorities focused on identifying sources of infection and tracing relevant contacts (containment) to prevent transmission and break chains of infection at an early stage, or better still, hinder their ‘emergence’ [3]. Despite the fact that vaccinations are now available, it is still important to know relevant transmission routes in order to contain the COVID-19- or further pandemics. Transmission primarily takes place when a household has a secondary attack rate (SAR) of 21.1% of infections [4]. Many different factors play a role here, including family size and the duration and intensity of contact [5]. Other scenarios include contact at the workplace [6], schools and daycare centres [7], nursing homes [8] and community shelters [9] and through direct patient care [10]. At the beginning of the pandemic, travellers returning home played a similarly prominent role [11]. Today, Infections via unknown transmission situations were problematic; in one Japanese study, for instance, these amounted to 32.9% of infections [12]. Possible infections in ‘anonymous’ settings such as supermarkets, restaurants or public transport can only rarely be detected; therefore, chains of infection cannot be reliably traced in these contexts. Meanwhile, other possible infection routes include aerosol transmission in rooms not containing other persons or communication via asymptomatic or presymptomatic carriers [13].

Based on the data of the public health department, Cologne, from February to December 2020, this study analyses possible transmission situations. The aim of this study was to gain a better understanding of transmission situations to enable a more targeted and rapid identification of infection routes. From today’s perspective, such knowledge can primarily contribute to pandemic containment if there is insufficient immunity due to lack of vaccination or as a result of immune escape variants.

## Methods

### Study design

In Germany, people are obliged to notify the local health authorities if COVID-19 infection is detected. Following this notification, the affected persons are called and questioned in a standardised manner to carry out targeted containment. The interviewers are trained to conduct a survey that records the suspected transmission situation and close contact persons in accordance with §16 of the ‘Infection Protection Act for the prevention of communicable diseases’. Based on the information provided by the interviewee, the assessment of transmission route was made taking into account the possible incubation period as well as factors such as distance, duration or protection. The results are documented with the assistance of a digital database system (‘Digitales Kontaktmanagement’ or DiKoMa) [14].

### Study population

**Fig 2:**
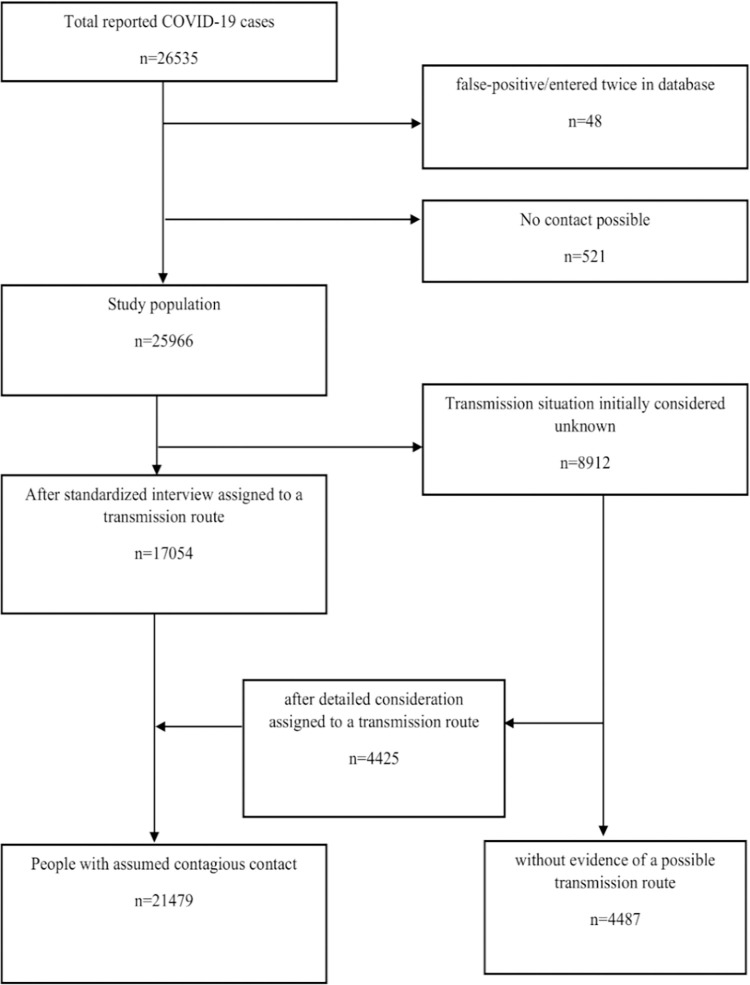
Flow chart: Study population.

The public health department of Cologne is responsible for infection prevention and control for 1.1 million inhabitants. Between 28 February 2020 and 31 December 2020, a total of 26,535 positive polymerase chain reaction (PCR) cases were reported. However, 48 of these persons were excluded due to false-positive test results or because they were entered twice into the database; meanwhile, 521 persons could not be contacted due to missing contact data. Therefore, 25,966 cases were integrated into this evaluation (see Fig 2 and Table 1, 51.4% female (n = 13,343)). The mean age was 41.6 +/- 20.5 years (median 39; IQR(0.25) = 26; IQR(0.75) = 55). Individuals were contacted a median of one day after performing their PCR test (mean 1.2 days; median 1 day; IQR(0.25) = 1 day; IQR(0.75) = 1 day). The proportion of individuals with no clear symptom onset was 27.9% (n = 7,249), and 395 persons (1.5%) died in or after the period under review in connection with their COVID-19 infection.

**Table 1:** Study population.

	Total	male	female
**Sample n (%)**	25,966	12,623 (48·6%)	13,343 (51·4%)
**Mean age (y; mean and SD)**	41·6 (20·5)	40·9 (19·9)	42·3 (21·0)
**Age Groups n (%)**			
0–2 y	236 (0·9%)	140 (0·5%)	96 (0·4%)
3–5 y	254 (1·0%)	113 (0·4%)	141 (0·5%)
6–10 y	604 (2·3%)	338 (1·3%)	266 (1·0%)
11–13 y	472 (1·8%)	267 (1·0%)	205 (0·8%)
14–17 y	902 (3·5%)	459 (1·8%)	443 (1·7%)
18–44 y	12,658 (48·7%)	6,093 (23·5%)	6,565 (25·3%)
45–64 y	7,312 (28·2%)	3,661 (14·1%)	3,651 (14·1%)
≥ 65 y	3,528 (13·6%)	1,552 (6·0%)	1,976 (7·6%)
Excluded	521	312	209

In this analysis, both reliably proven infection routes and the most probable sources of infection were considered, taking into account the course of infection and medical history.

### Survey

The following data were recorded:

- the symptomatology and symptom onset and/or positive test date;
- the patient’s address and information on the suspected transmission situation;
- if applicable, a known infected person from the patient’s environment (source of infection);
- the patient’s employer or educational institution, community facilities visited, and hospitalisation.

### Categorisation of known transmission situations

The most frequently described transmission situations were identified based on a literature analysis and categorised as follows (for an overview, see Table 2): social contact; work contact; travel return (within 14 days before the onset of the disease); care facilities for the elderly, youth and disabled; educational institutions; community accommodation; inpatient or outpatient medical treatment; and unknown. Additionally, the workplaces were catagorised to different sectors. Children attending educational institutions were assigned to the corresponding type of school, and known cases at a residential address within 14 days after infection were identified.

**Table 2:** Classification of transmission situations.

Transmission category	Detailed transmission route	
Social contact	Close contact	Household transmission
		Partners
		Family members
	Distant contact	Non-family members
		Parties
		Religious institutions
		Sport (indoor/outdoor)
		Restaurants
		Choir
		No detailed data
Workplace contact	High-risk occupations	Medical care
		Nursing
		Education
		Retail staff
	Other	Other
Travel return		
Care facilities	Inpatient care facilities for the elderly, youth and disabled	
	Outpatient geriatric care services	
Educational institutions	Daycare centres	
	Schools	
Infections in community settings	Prisons	
	Monasteries	
	Refugee accommodation	
Infections in the medical sector, including nursing in hospitals		
Unknown infection after detailed assessment		
Excluded: Case not reached for information		

### Assessment of initially unknown transmission situations

Persons whose transmission situation was initially considered unknown (n = 8,912; 34.3%) were contacted and questioned again in detail. Known cases in their environment (e.g., workplace, school, daycare centre, common residential address) immediately before infection (maximum 14 days) were considered to clarify the transmission situation. Subsequently, those with a high risk of infection at the workplace, such as those involved in patient care, nursing staff, and staff in schools and daycare centres, were assigned to this infection route. Meanwhile, children who attended educational institutions during the period in question were assigned to this transmission situation last if no other indications were available.

### Data processing

Transmission situations were analysed using SPSS 27.0 (IBM, Armonk, NY, USA). Means, standard deviations, medians, IQR and frequencies were calculated for this purpose. Associations between categorical variables, such as symptomatic (yes/no) and transmission situation (known/unknown), were examined using an χ2 test. The significance level was set at α = 0.05. Lastly, to illustrate the course of the pandemic, the seven-day incidence/100,000 population and the reproduction number Rt at a generation time of four days were calculated.

### Ethical considerations

An ethical vote and a data protection impact assessment on the use of the data, which were primarily collected for targeted containment and for possible renewed contact with the persons concerned, were made available from the City of Cologne and the RWTH Aachen Human Ethics Research Committee (File No 351/20).

## Results

### Transmission situations

Initially, 65.7% of the transmission situations could be categorised, while the second analysis/second survey yielded a probable infection situation for a further 4,425 persons. Thus, a transmission situation could not be identified for a total of 4,489 cases (17.3%). These individuals were slightly more likely to be male (n = 2,427; 54.1%) and older (mean 46.5 +/- 18.4 years) than the total study population.

**Fig 3:**
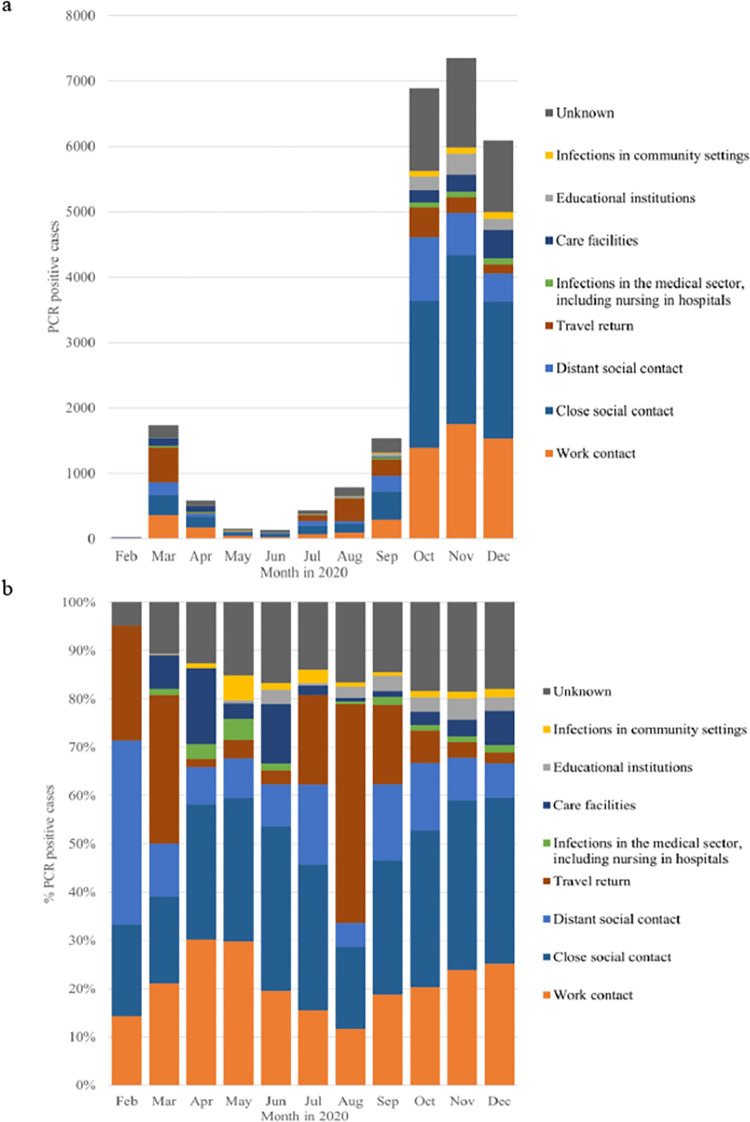
Different transmission routes over the year 2020 (a and b).

An assumed contagious contact could be determined for 82.7% (n = 21,477) of the persons contacted (min 81.6%; n = 5,997 in November 2020; max 95.2%; n = 20 in February 2020). The proportions of infection situations by age are shown in Table 3 and Fig 3. In persons without a clear onset of symptoms, the route of infection was determined significantly more often (n = 6,134; 84.6%) than in symptomatic persons (n = 15,343; 82.0%; p<0.001). Moreover, in asymptomatic persons, the proportion of transmission situations differed slightly from the total sample. In particular, the proportion of infections among nursing home residents, at 10.8% of the asymptomatic cases, was considerably higher than in the total sample (4.4%). There were also differences in the proportion of asymptomatic individuals among minors. Indeed, 39.8% (n = 658) of children up to 17 years old who were infected through social contact did not show any symptoms. This proportion was significantly lower in the same age group among those infected at educational institutions (23.9%; n = 138; p<0.001).

**Table 3:** Transmission routes at different ages.

	**0–2 years**		**3–5 years**		**6–10 years**		**11–13 years**		**14–17 years**	
Social contact	174	73·7%	171	67·3%	425	70·4%	320	67·8%	562	62·3%
Workplace contact	0	0·0%	0	0·0%	0	0·0%	0	0·0%	13	1·4%
Travel return	10	4·2%	14	5·5%	29	4·8%	24	5·1%	30	3·3%
Care facilities	0	0·0%	0	0·0%	3	0·5%	1	0·2%	6	0·7%
Educational institutions	33	14·0%	56	22·0%	123	20·4%	108	22·9%	257	28·5%
Infections in community settings	6	2·5%	11	4·3%	22	3·6%	17	3·6%	29	3·2%
Infections in the medical sector	0	0·0%	0	0·0%	0	0·0%	0	0·0%	2	0·2%
Unknown infection	13	5·5%	2	0·8%	2	0·3%	2	0·4%	3	0·3%
Excluded	3		1		2		3		9	
Total (without excluded)	236		254		604		472		902	
	**18–44 years**		**45–65 years**		**>65 years**				**Total**	
Social contact	5,169	40·8%	2,952	40·4%	1,158	32·8%			10,931	42·1%
Workplace contact	3,531	27·9%	2,143	29·3%	104	2·9%			5,791	22·3%
Travel return	1,224	9·7%	628	8·6%	127	3·6%			2,086	8·0%
Care facilities	59	0·5%	98	1·3%	1,024	29·0%			1,191	4·6%
Educational institutions	206	1·6%	5	0·1%	0	0·0%			788	3·0%
Infections in community settings	195	1·5%	42	0·6%	24	0·7%			346	1·3%
Infections in the medical sector	31	0·2%	59	0·8%	252	7·1%			344	1·3%
Unknown infection	2,243	17·7%	1,385	18·9%	839	23·8%			4,489	17·3%
Excluded	285		134		84				521	
Total (without excluded)	12,658		7,312		3,528				25,966	

### Social contact

Social contact was responsible for 42.1% (n = 10,931) of the reported infections throughout 2020. In the group of minors, transmissions occurred more frequently as a result of social contact (0–17 years; 66.9%) than in the group of professionals (18–64 years; 40.7%). In the age group of ≥ 65 years, the proportion of transmissions resulting from social contact decreased to 32.8%.

A total of 54.0% (n = 5,903) of transmissions occurred within cohabiting households, whereas 41.5% (n = 4,530) occurred outside cohabiting households. The majority of transmissions outside households were among family members and life partners (n = 2,339).

### Workplace contact

Workplace contact accounted for a total of 22.3% of infections (n = 5,791). Meanwhile, if only the age group 18–64 was considered, the proportion of transmissions via work contact was 28.4% (n = 5,674).

The most frequent transmissions (57.9%) occurred in the following three areas: inpatient nursing homes or outpatient care (outside hospitals), medical staff (including nurses in hospitals), and educational institutions. Retail staff constituted 2.6% of work contact infections (n = 151) and staff at restaurants and bars for another 3.0% (n = 173). Women were significantly more likely to be infected through contact at work (55.7%; n = 3,219) than men (p<0.001), likely because they were more prevalent in the high-risk occupations of education, nursing and medical care.

### Educational institutions

In the entire period, there were 788 infections (3.0%) resulting from attending an educational institution, such as a daycare centre, primary school, special school, secondary school or vocational school. Together with occupational infections in the field, a total of 6.9% (n = 1,801) of infections occurred in the context of educational institutions (see Table 4).

**Table 4:** Number of infections in educational institutions.

	Mean age (y; mean and SD)	visitors	employees	ratio
**daycare centre**	3·4 +/- 1·6	94	335	1:3·6
**primary school**	8·0 +/- 1·2	93	230	1:2·5
**secondary school**	14·6 +/- 2·3	355	245	1:0·7
**Special needs school**	13·1 +/-3·2	25	80	1:3·2
**vocational school**	21·9 +/- 6·8	218	102	1:0·5

In the group of people younger than 18 years, only 23.4% (n = 577) were infected in educational institutions. Most persons in this age group became infected during social contact within their household (49.6% of 0–17-year-olds with a known transmission situation; n = 1,225).

### Care facilities

Overall, infections in care settings accounted for 8.2% (n = 2,127) of the total. These included inpatient care facilities for the elderly and disabled, inpatient youth care facilities and outpatient geriatric care services. Broken down by individual settings, there were a total of n = 1,152 (4.4%) infections of residents in nursing homes. The majority were related to care for the elderly.

Only 33.9% (n = 944) of people older than 70 years were infected in nursing homes. Thus, the majority of transmissions in this age group also took place outside care facilities. Only 39 transmissions (0.2%) occurred in outpatient care services.

### Infections in the medical sector, including nursing in hospitals

1.3% of infected persons (n = 344) were patients who became infected in the outpatient or inpatient medical sector. Infections in the medical sector, including employees and patients, constituted 6.8% (n = 1,758) of total infections over the entire period.

### Infections in community settings

Outbreaks in community settings had a low impact (n = 346; 1.3%) and primarily involved outbreaks in refugee accommodation.

### Travel return

**Fig 4:**
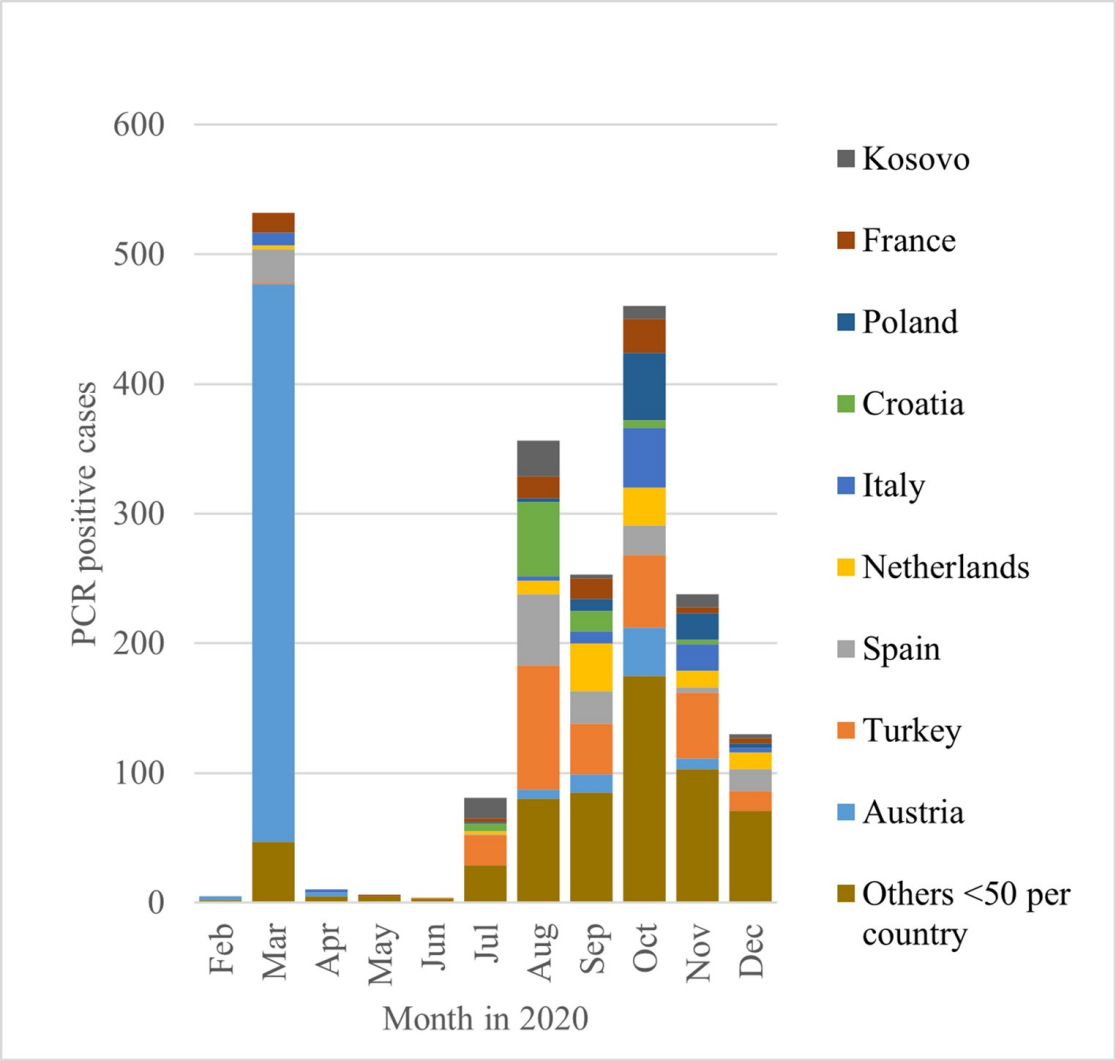
Infections after travel return.

In the entire period (from March to December 2020), travel returnees accounted for only 8.0% (n = 2,086) of infections. However, significant increases were evident at the beginning of the first and second waves. In March, 30.7% of the cases were travel returnees (n = 532). Holidaymakers from ski resorts, primarily in Austria, initially played a decisive role, particularly as an obligation to quarantine (or present a negative test) after entering Germany from risk areas did not exist until 10 April 2020 [15]. During and after the summer holidays, there was a renewed increase in such cases: in August, for instance, this group accounted for 45.4% (n = 356) of infections. At that time, there was an obligation to quarantine (or present a negative test) when entering Germany from outside the European Union or from risk areas within it. Nevertheless, the majority of positive cases came from countries that did not fall under these criteria at the time (see Fig 4).

## Discussion

Following a survey and literature-based assessment, more than 80% of the infections reported to the Cologne health department could be assigned to a plausible transmission situation between February and December 2020. In this way, approximately half of the infection situations initially assessed as unknown in origin could subsequently be detected. With regard to the remaining unknown cases, it is conceivable that they were transmitted via close contacts whose infection was not (yet) known or who had not reported their infection for fear of possible consequences (e.g. for attending unauthorised events). Also conceivable are anonymous settings in which the route of infection cannot be traced, such as on public transport, in supermarkets or in empty rooms potentially contaminated with infectious aerosols. However, based on our findings, these anonymous settings played only a minor role, suggesting that previously employed protective measures mainly implemented until then, such as mask-wearing and social distancing, were sufficient.

Among the known infection routes, social contact played by far the most prominent and most continuous role, while the duration of contact was highly influential. Indeed, Thompson et al. [4] showed that social contact led to high transmission rates, particularly if the period of this contact was longer than five days, which is assumed in the case of household members. Social contact in the context of social events (such as family celebrations) was more significant (with a SAR of 5.9%) than simple social contact, such as that with strangers or casual contact (1.2%).

Although work contact accounted for about a quarter of the known infections, this was challenging to summarise under one SAR due to the heterogeneity of possible situations. The areas of medical care (outpatient and inpatient), outpatient and inpatient care (outside hospitals) and education (school and daycare) were particularly significant. These contexts accounted for more than half of the infections through contact at work. Infections of staff in nursing or medical care were particularly notable in the first wave, perhaps due to the insufficient supply of protective equipment at the beginning of the pandemic due to the decrease of this infection route during the observation period [16]. This further underlines the compulsory vaccination in health professions, which was introduced in Germany in March 2022. Besides contacts with many patients the majority of transmissions in workplaces occurred through children. In the education sector, the officially ordered closures of facilities and school holidays were reflected in lower transmissions. Nonetheless, a shift in the transmission ratio in different age groups also became apparent. Whereas in daycare, more educators than children tested positive, this was reversed in secondary schools. A SAR that increases with age has already been described in various studies, and children presumably become more susceptible to the virus with increasing age [17]. In addition, our data showed that children infected in schools were more likely to be symptomatic than those infected by a household member. Many infections in education settings were, therefore, likely overlooked due to the lack of symptoms and/or regular testing, an inference also indicated in a screening study in which the number of antibody-positive children was six times higher than the number of reported cases [18]. It could thus be assumed that a higher proportion of detected asymptomatically infected persons in the surroundings of individuals who have tested positive would result in a lower number of unreported cases. Care facilities are another example of an environment wherein an above-average number of asymptomatically infected persons was detected. As such, through standardised regular serial testing, many infected persons could be found in this context regardless of the presence or absence of symptoms. Literature shows that daily antigen testing, for example in nursing home staff, has the greatest benefit in reducing covid-19 outbreaks. Immunity to Sars-CoV-2 (through infection or latter vaccination) among staff has also had a major effect in preventing outbreaks in studies [19]. Immunity as a prerequisite for working in high-risk areas and regular antigen testing could significantly reduce infections in these situations. The same could be applied in educational institutions. An alternative method for detecting infections in children to reach a higher specificity and sensitivity would be pooled RT-qPCR [20]. Since younger children, in particular, will only be able to be vaccinated in the distant future, there is a specific focus here on alternative infection control measures. For this purpose, we recommend (mandatory) vaccination for teachers and caregivers and the maintenance of hygiene measures to prevent outbreaks in schools and daycare centres. The recommendation for vaccination and immunisation in high-risk areas is dependent on the effectiveness of vaccination against spread of infection in different subtypes of Sars-CoV-2. The study was conducted predominantly during circulation of the wild type and alpha variant. However, especially in the first months after immunisation, good protection against infection and thus transmission is also observed with the omicron variant [21].

Although infections in care facilities or during medical treatment account for a small proportion of total transmissions (n = 1,535; 5.9%), these two transmission situations were responsible for more than 50% of deaths. Preventing outbreaks in these situations through (mandatory) testing and protecting employees has great potential to prevent deaths with minimal effort.

In absolute terms, infections in other community facilities occurred less frequently during the pandemic. However, such facilities are generally more affected by outbreaks; for example, in refugee shelters, social distancing and hygiene rules are difficult or impossible to implement. The extent to which adequate accommodation in smaller cohorts or quarantine hotels or even compulsory vaccination can be recommended remains open to debate.

Infections from abroad only played a relevant role in selected periods. It can be assumed that vaccinations led to considerable shifts here. Nevertheless, imported infections from abroad played a decisive role at the beginning of the pandemic. Since new virus variants are still emerging for which vaccination does not offer the same protection as at the beginning, the existing regulations should be adhered to [22, 23].

### Strength and limitations

This study is an overall survey and systematic analysis of the transmission situation and contact data of infected persons in Cologne, the fourth-largest city in Germany. While the study has contributed important insights concerning transmission sources, the research has some limitations. Firstly, the information of transmission situation provided by the interviewees could not be verified (that is, the truth of the content could not be assessed). This issue may have resulted in the underreporting of infection situations that were legally prohibited at the time, such as private parties. Secondly, as the number of cases increased, there was a delay in making contact, and, as a result of these dynamics, it may no longer have been possible to enquire in detail about all transmission situations. Thirdly, the survey period is of utmost importance. Since it was before the availability of possible vaccines, the data are not necessarily transferable from today’s perspective. However, it is precisely this that offers the opportunity to draw conclusions for new pandemics. During the study period, the wild-type variant was dominant. Regular sequencing to identify variants did not begin until mid-January 2021. Although shifts in the distribution of infection routes due to the emergence of more infectious variants and/or an increasing number of (fully) vaccinated individuals can be assumed, these factors could not be considered in this work. Vaccinations started at the end of 2020, regular sequencing to identify variants began mid-January 2021. Moreover, as Omicron as the current leading strain has a higher immune escape, anyway recommendations in terms of protective measure, including vaccination are of limited evidence. Nevertheless, parallels can be seen in the current waves, for example, with regard to the high proportion of travellers returning at the beginning of waves.

## Conclusion

This study has shown that myriad previously unknown infection situations can be elucidated through precise documentation and comprehensive analysis. Results suggest that infections mainly occur in the family sphere, at the workplace or at school. Regarding the workplace, most infections occur in professions that demand close contact, such as care for the elderly, and entail a high risk of exposure, such as work in hospitals. Besides vaccination it is strongly recommended, therefore, to carry out regular and systematic testing in areas or occupations with a high risk of transmission or contact with vulnerable groups of people, in schools, among household members of an infected person and of people arriving from other countries.
