# Correction: Where is the greatest risk of COVID-19 infection? Findings from Germany’s largest public health department, Cologne

**DOI:** 10.1371/journal.pone.0305302

**Published:** 2024-06-06

**Authors:** Lukas Broichhaus, Julian Book, Sven Feddern, Barbara Grüne, Florian Neuhann, Johannes Nießen, Gerhard A. Wiesmüller, Annelene Kossow, Christine Joisten

In the Author Contributions, Lukas Broichhaus should be listed as the only author who wrote the paper under ’Writing–original draft.
